# Quantitative Comparison of the Clinical Efficacy of 6 Classes Drugs for IgA Nephropathy: A Model-Based Meta-Analysis of Drugs for Clinical Treatments

**DOI:** 10.3389/fimmu.2022.825677

**Published:** 2022-03-28

**Authors:** Jiesen Yu, Jieren Luo, Haoxiang Zhu, Zichao Sui, Hongxia Liu, Lujin Li, Qingshan Zheng

**Affiliations:** Center for Drug Clinical Research, Shanghai University of Traditional Chinese Medicine, Shanghai, China

**Keywords:** IgA nephropathy, model-based meta-analysis, urinary protein, corticosteroids, immunosuppressants, N-3 fatty acids

## Abstract

**Introduction:**

There is a wide variety of drugs for the clinical treatment of immunoglobulin A (IgA) nephropathy; however, previous studies have failed to clarify the quantitative differences in the efficacy of various drugs. In this study, we aimed to quantitatively compare the clinical efficacy of 6 classes of drugs with different pharmacological mechanisms for the treatment of IgA nephropathy and to identify relevant influencing factors.

**Methods:**

Clinical trials of drugs for the treatment of IgA nephropathy were obtained from public databases. The change in daily urinary protein excretion from baseline was used as the efficacy index, and the time–effect model was established using a model-based meta-analysis method. Based on the final model, the typical efficacy was simulated, and the differences in efficacy were compared.

**Results:**

A total of 40 studies with 2288 subjects were included in this study. The results showed that the time–effect relationship of the placebo and 6 classes of drugs was consistent with the E_max_ model. The placebo reduced urinary protein excretion by up to 0.44 g/day, and it took more than 27 months to reach half of its maximum effect. The onset of the 6 classes of drugs were the same; they all reached half of their maximum effect after 5.59 months. More importantly, we found a significant influence of urinary protein baseline on drug efficacy, as indicated by an increase of 0.63 g/day in the theoretical maximum effect of drugs for every 1 g/day increase in urinary protein baseline. After correcting for the urinary protein baseline, the order of efficacy of the 6 classes of drugs was as follows: corticosteroids > immunosuppressants > other drugs > renin–angiotensin system blockers > antiplatelet agents > N-3 fatty acids.

**Conclusion:**

This study provides the first comprehensive quantitative analysis of the differences in the efficacy of 6 classes of drugs with different pharmacological mechanisms for treating IgA nephropathy. The results of this study provide an important reference for the rational clinical use of drugs for IgA nephropathy, and also provide a reliable efficacy standard for the development of new drugs for IgA nephropathy.

## Introduction

Immunoglobulin A (IgA) nephropathy is a primary glomerular disease characterized by the deposition of IgA in the mesangial region (1, 2), and 10–20% of patients with IgA nephropathy develop end-stage renal disease within 10 years (3). Disease progression is often accompanied by a variety of complications, such as glomerulosclerosis, renal interstitial fibrosis, hypertension, proteinuria, and renal insufficiency (4, 5).

At present, the clinically available drugs for the treatment of IgA nephropathy are divided into 5 categories based on their pharmacological mechanism (6–8): renin–angiotensin system (RAS) blockers, corticosteroids, immunosuppressants, antiplatelet agents, and N-3 fatty acids. Although the guidelines issued by Kidney Disease Improving Global Outcomes (KDIGO) in 2021 and the Japanese Society of Nephrology (JSN) in 2014 provide the recommended levels for clinical treatment involving various drugs, the difference in treatment efficacy among different drugs is unclear due to the lack of systematic evaluation, which makes it difficult for clinicians to standardize the use of drugs. In 2018, Yang et al. compared the efficacy of several commonly used treatment regimens for IgA nephropathy using network meta-analysis and revealed that the combination of RAS blockers and glucocorticoids has the best efficacy (9). However, this study only compared 6 regimens, which does not fully reflect the treatment status of IgA nephropathy. In addition, due to the limitations in the network meta-analysis method, this study only analyzed the efficacy at the end point (the treatment course ranged from 3 to 120 months) and failed to exclude the effect of heterogeneity in the treatment course on the results.

Model-based meta-analysis (MBMA) is a combination of mathematical modeling and meta-analysis, which quantitatively evaluates the time-course characteristics and influencing factors of drug efficacy and provide necessary pharmacodynamic parameters, such as maximum efficacy and onset time (10, 11). The purpose of this study was to quantitatively compare the differences in the efficacy of different classes of drugs for the treatment of IgA nephropathy using MBMA and to identify relevant influencing factors to provide valuable information for the clinical guidelines of IgA nephropathy.

## Method

### Search Strategy and Inclusion Criteria

A literature search was conducted using the PubMed and Embase databases to collect clinical trials related to IgA nephropathy conducted before November 18, 2019. The search keywords included indications and drug names; the logical operator “AND” was used for different categories of keywords, and the logical operator “OR” was used for similar keywords. The detailed search strategy is provided in the **Table S1**.

The KDIGO 2021 clinical practice guideline for the management of glomerular diseases and the individual participant meta-analysis conducted by Inker LA et al. in 2021 indicated that urinary protein is an effective indicator to measure the progress of IgA nephropathy (12, 13). The change in daily urinary protein excretion (g/day) from baseline was an indicator of IgA nephropathy in this study. References meeting the following inclusion criteria were included in the analysis: (1) clinical trials, (2) adult patients with IgA nephropathy were included as the subjects, (3) daily excretion of urinary protein was being reported, and (4) the report was written in English.

### Data Extraction

Microsoft Excel (2016 edition) was used to extract the following information: (1) Literature features: first author, year of publication, region of publication, etc.; (2) Test design: test drug, course of treatment, sample size, etc.; (3) Characteristics of subjects: age, weight, proportion of male subjects, proportion of white subjects, baseline level, and so on; and (4) Test results: the change in daily urinary protein excretion from baseline at each time point. Data were extracted independently by two researchers, and disputes were resolved through discussions with a third researcher. If the data in the literature were presented graphically, Engauge Digitizer software was used to extract the data from the graphics.

### Model Building

The data distribution of the change in daily urinary protein excretion from baseline was consistent with the classic pharmacodynamic model E_max_ model (Equation 1) (14, 15); the efficacy gradually increased with time and eventually reached the efficacy plateau.


(1) $$E_{i,j}=\frac{E_{\max,i}\times Time_{i,j}}{ET_{50,i}+Time_{i,j}}$$

In Equation 1, E_i, j_ is the efficacy of the i^th^ drug or placebo arm at time point j. E_max,i_ is the theoretical maximum efficacy of the i^th^ drug or placebo arm, and ET_50,i_ is the time required to reach half of E_max,i_, reflecting the onset speed of the drug or placebo.

The differences in drug or placebo effects among different studies may be expressed by inter-study variability (η) (16). In this study, the scale model was used to describe inter-study variation (Equation 2):


(2) $$P_{i}=P_{typical}\times (1+\eta_{i})$$

In Equation 2, P_i_ is the value of the model parameter of the i^th^ drug or placebo arm, P_typical_ is the typical population value of the model parameter, and η_i_ is the variation in the model parameter between studies, conforming to a normal distribution with a mean of 0 and variance of ω^2^.

Unexplainable variation was classified as residual error equation(ϵ). In this study, the addition model was used to explain the residual error variation (Formula 3):


(3) $$Y_{observe,i,j}=Y_{predict,i,j}+\frac{\epsilon_{i,j}}{\sqrt{N_{i,j}}}$$

In Equation 3, Y_observe,i,j_ is the observed efficacy of the i^th^ drug or placebo arm at the j^th^ time point, Y_predict,i,j_ is the predicted efficacy of the i^th^ drug or placebo arm at the j^th^ time point, and ϵ_i,j_ is the residual error of arm i at the j^th^ time point, which must be corrected for the sample size (N_i,j_). That is, a larger sample size is associated with a smaller residual error. ϵ_i,j_ conforms to a normal distribution with a mean of 0 and variance of σ^2^ (17).

Covariate models were used to examine potential factors affecting model parameters, including age, sex, weight, race, urinary protein excretion at baseline, and drug class. If the missing rate of a covariate was less than 30%, the missing data were replaced with the median value of the remaining data; if the missing rate of a covariate was greater than 30%, the covariate was not investigated.

The way a covariate was introduced into the model depended on the data type of the covariate. Categorical variables were introduced using Equation 4, and continuous variables were introduced using Equations 5–6.


(4) $$P_{\text{i}}=P_{\text{pop}}+COV_{i}\times \theta_{COV}$$

(5) $$P_{\text{i}}=P_{\text{pop}}+(COV_{i}-COV_{median})\times \theta_{COV}$$

(6) $$P_{\text{i}}=P_{\text{pop}}\times {\left(COV_{i}/COV_{median}\right)}^{\theta_{COV}}$$

In Equations 4–6, P_i_ is the individual value of the model parameter of the i^th^ drug or placebo arm, and P_pop_ is the typical population value of the model parameter P when the categorical covariate is equal to 0 or continuous covariate is equal to COV_median_. θ_cov_ is the correction coefficient of the covariates for the model parameters. COV_i_ represents the covariate value of the i^th^ study, and COV_median_ represents the median of the covariates in the analysis dataset.

First, the forward inclusion method was used to investigate the influence of each covariate on the efficacy parameters. If the objective function value of the model decreased by more than 3.84 (degree of freedom, 1; P = 0.05 chi-square distribution boundary value), the covariate was considered to have a significant influence on the parameters. All the covariates found to have significant influences through individual screening were screened again through the backward elimination method to confirm that the covariates finally entered into the model. The bound value of backward elimination was 6.63 (P < 0.01) (18, 19).

### Model Evaluation

First, the performance of the model was evaluated using the model diagnostic goodness-of-fit plots. Second, the bootstrap method was used to evaluate the stability of the model. Finally, Visual Predictive Check was used to compare the degree of agreement between the predicted and observed values of the model and evaluate the prediction performance of the model (20–22).

### Model Prediction

Based on the typical values of the model parameters, the 95% CIs of the typical efficacy values of each drug under different conditions were calculated using Monte Carlo simulation 1000 times. If the 95% CIs of the typical efficacy values of the two drugs did not overlap, this indicated that the two drugs exhibited significant differences in efficacy. The order of efficacy of various drugs was determined by ranking the efficacy values.

### Software

Data modeling and simulation were performed using NONMEN 7.4 (Level 1.0, ICON Development Solutions, USA). R software (Version 3.6.1) was used to sort the output data, statistical analysis, and plotting.

## Results

### Characteristics of the Included Studies

**Figure 1:**
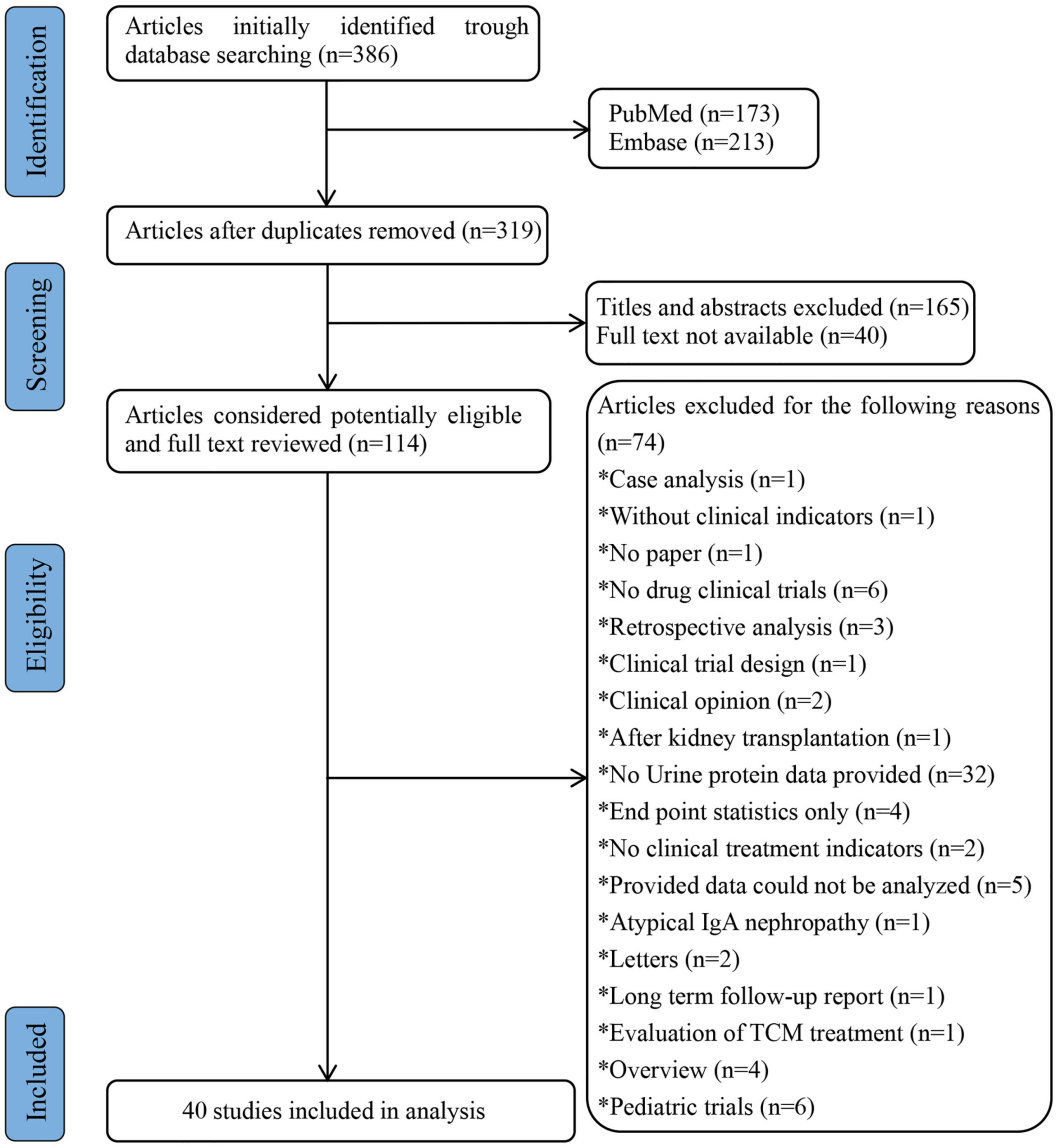
Flow chart of literature screening.

Finally, a total of 40 studies with 2288 subjects were included in the analysis, and the literature screening process is shown in **Figure 1**. A total of 14 studies (419) were included in the placebo group, and 39 studies (1869) were included in the drug group. The included studies in the drug group were classified into 6 classes with different pharmacological mechanisms according to the classification criteria of the clinical trial summary in the evidence-based clinical practice guidelines for IgA nephropathy published by the JSN in 2014. The 6 classes of drugs included “corticosteroids”, “immunosuppressants”, “RAS blockers”, “antiplatelet agents”, “N-3 fatty acids” and “other drugs”. The treatment drugs that do not fall into the top 5 classes and the combination of drugs in different categories were classified into the “other drugs”. The included studies were published between 1987 and 2017, and the duration of clinical trials ranged from 1 to 48 months. Detailed information on the included studies is presented in **Table 1**.

**Table 1:** Characteristics of the included literature.

	Overall	All Drug	Placebo	Corticosteroids	Immunosuppressant	RAS blockers	Antiplatelet agents	N-3 fatty acids	Other drugs
Number of trials/arms	40/83	39/69	14/14	8/9	11/13	18/27	2/2	4/5	10/13
Total sample size	2288	1869	419	288	415	634	28	157	347
Sample size per arm, Median (Min, Max)	20 (6, 114)	20 (6, 112)	19 (6, 114)	21 (6, 106)	21 (11, 101)	15 (6, 112)	14 (8, 20)	36 (14, 55)	20 (9, 48)
Baseline, g/day, Median (Min, Max)	1.9 (0.57, 5.29)	1.9 (0.57, 5.29)	1.86 (0.73, 4.57)	1.6 (0.57, 2.14)	2.77 (1.35, 5.29)	1.72 (0.6, 2.48)	0.83 (0.73, 0.92)	1.79 (1.31, 2.55)	2.1 (0.94, 3.7)
Age, year, Median (Min, Max)	37 (24.7, 52)	37 (24.7, 52)	37 (27.5, 52)	33.8 (24.7, 40.5)	37 (32.5, 43)	37 (27.8, 52)	35.15 (33.3, 37)	41 (37, 46)	34 (25, 50)
Gender, male%, Median (Min, Max)	58.33 (11.11, 94.12)	58.33 (11.11, 94.12)	58.33 (30.91, 86)	61.9 (11.11, 77)	58.33 (36.36, 94.12)	57.14 (24.07, 81.82)	35.42 (12.5, 58.33)	74 (46.6, 84)	60 (33.33, 85.71)

### Model Building and Evaluation

In this study, the influences of the daily urinary protein excretion at baseline, age, and sex ratio on model parameters were investigated, and it was found that the daily urinary protein excretion at baseline had a significant influence on E_max_ of the drug arm. With each increase in daily urinary protein excretion from baseline of 1 g/day, E_max_ of the drug arm increased by 0.63 g/day, as shown in Equation 7.


$$E_{max,drug,i}=E_{max,drug,typical}-(Baseline-1.82)\times 0.63$$

The daily urinary protein excretion at baseline had no significant effect on E_max_ of the placebo arm. In addition, because the ET_50_ estimates of different drug arms were similar, to simplify the model, 6 classes of drugs with different pharmacological mechanisms shared the same ET_50_ value. The estimated η(E_max_) value was similar to 0; therefore, considering the stability of the model, the η(E_max_) value was finally fixed at 0. The final model parameter estimations are presented in **Table 2**.

**Table 2:** Estimated values of final model parameters and Bootstrap resampling parameters.

Parameters	Mean Estimate	RSE(%)	95%CI	Bootstrap Median	Bootstrap 95%CI
E_max_, placebo, g/day	-0.44	38.10	-0.77 to -0.11	-0.44	-0.95 to -0.10
ET_50_, placebo, month	27.20	32.50	9.87 to 44.53	25.70	10.30 to 99.40
Baseline on E_max_, g/day	-0.63	18.30	-0.85 to -0.40	-0.63	-0.91 to -0.36
E_max_, Corticosteroids, g/day	-1.47	3.80	-1.58 to -1.36	-1.45	-1.72 to -1.26
E_max_, Immunosuppressant, g/day	-1.40	7.80	-1.61 to -1.19	-1.38	-1.72 to -1.06
E_max_, RAS blockers, g/day	-0.95	12.00	-1.17 to -0.72	-0.92	-1.28 to -0.72
E_max_, Antiplatelet agents g/day	-0.65	18.20	-0.88 to -0.42	-0.67	-0.92 to -0.39
E_max_, N-3 fatty acids, g/day	-0.53	35.30	-0.90 to -0.16	-0.54	-1.24 to -0.24
E_max_, Other drugs, g/day	-1.31	12.90	-1.64 to -0.98	-1.31	-1.74 to -0.95
ET_50_, Drug, typical, month	5.59	28.40	2.48 to 8.70	5.15	2.65 to 9.35
η (E_max_)	0 FIXED	–	–	–	–
η (ET_50_)	0.65	7.40	0.56 to 0.75	0.68	0.55 to 0.90
ϵ	1.54	8.00	1.30 to 1.78	1.48	1.16 to 1.75

**Figure 2:**
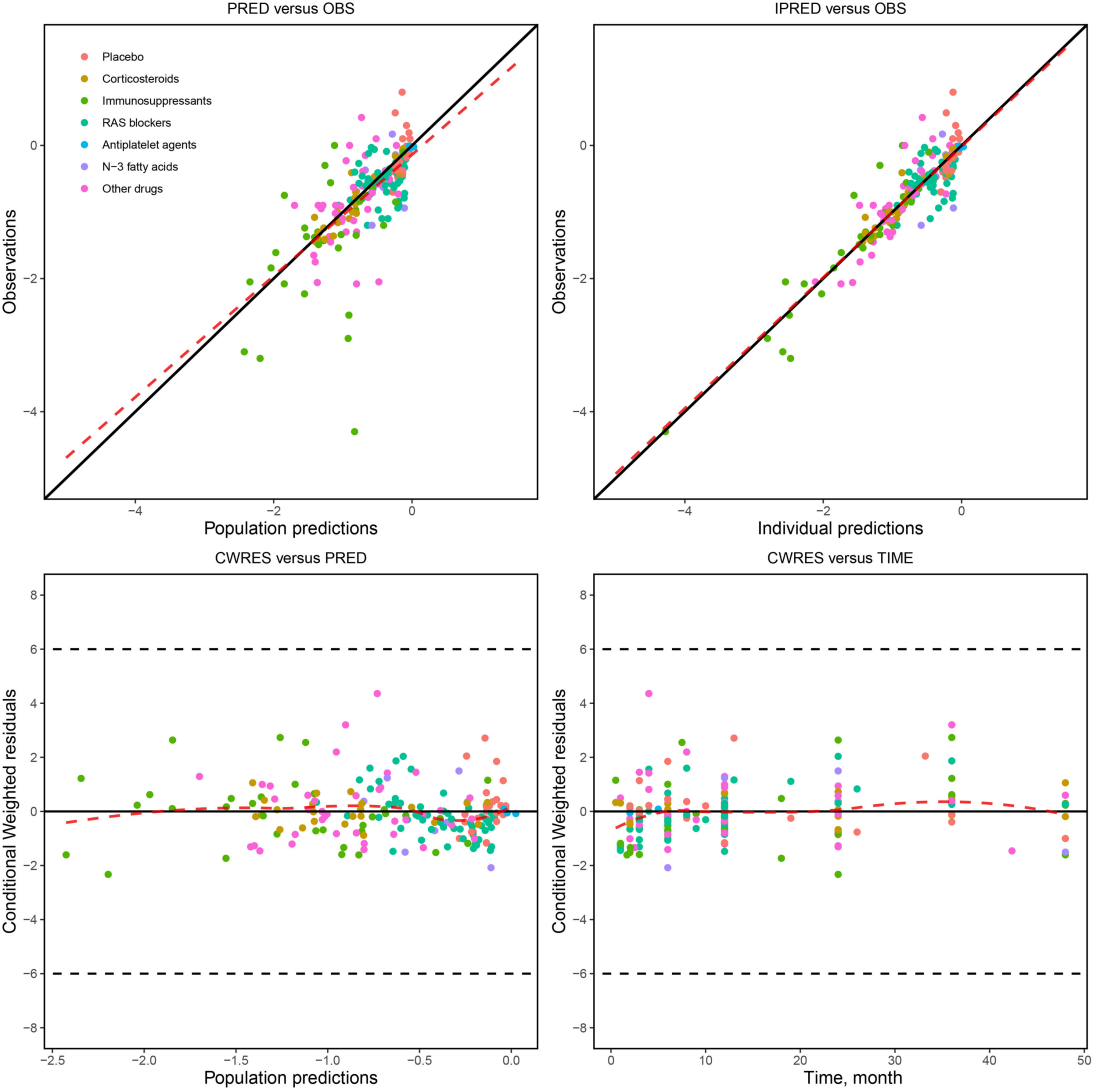
Model diagnostic goodness-of-fit plots.

The model diagnostic goodness-of-fit plots (**Figure 2**) show that the observed values (OBS) and population predicted values (PRED) and OBS and individual predicted values (IPRED) are evenly distributed on both sides of the diagonal, and the fitting line nearly coincides with the diagonal. The conditional weighted residual errors (CWRES) of most points are distributed evenly approximate to the 0 line within 4, and the fitting lines of CWRES vs PRED, and CWRES vs time, nearly coincide with the 0 line. These results show that the model has a good fit with the observed values without any obvious bias. The distribution of model parameters obtained using the bootstrap method was approximate to the estimated values of the model parameters obtained from the original dataset (**Table 2**), and the success rate of the bootstrap method for 1000 times was 98.1%, indicating that the estimation of model parameters was relatively stable and less affected by individual studies.

**Figure 3:**
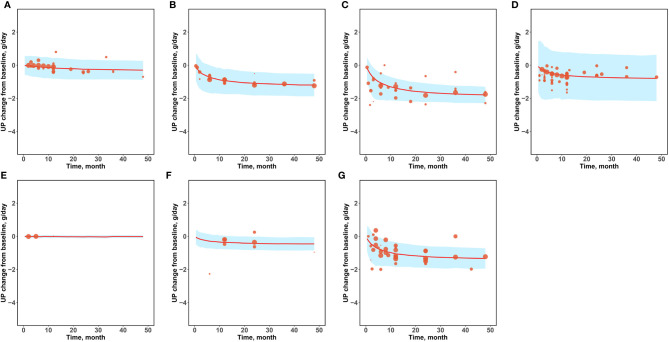
Prediction-corrected visual predictive check. **(A)** Placebo **(B)** Corticosteroids **(C)** Immunosuppressants **(D)** RAS blockers **(E)** Antiplatelet agents **(F)** N-3 fatty acids **(G)** Other drugs.

Since the daily urinary protein excretion significantly influences the baseline of the E_max_ value of the drug arm, and there is a large range of daily urinary protein excretion in this study (0.57–5.29 g/day), we conducted a prediction-corrected visual predictive check analysis (**Figure 3**) to correct for different baselines in order to accurately reflect the prediction performance of the model for drug efficacy at different baseline levels. The results after the baseline correction are shown in **Figure 3**. The 95% CIs predicted by the model in different subgroups covered most of the observed values, indicating that the model had a good predictive performance.

### Typical Efficacy of Placebo

Since the placebo group had a maximum effect of 0.44 g/day and had a slow onset, requiring more than 27 months to reach half of its maximum effect, the placebo efficacy in the IgA nephropathy clinical trial was low.

Model simulations showed that the typical placebo efficacies at 6 months, 1 year, 2 years, and 4 years were -0.08 (95% CI: -0.16, -0.03), -0.14 (95% CI: -0.25, -0.04), -0.21 (95% CI: -0.36, -0.06), and -0.28 (95% CI: -0.47, -0.08) g/day, respectively. The placebo efficacy at 4 years was approximately 2 times that at 1 year. In addition, the placebo efficacy was not affected by the daily urinary protein excretion at baseline and was comparable between patients with mild to moderate proteinuria and those with severe proteinuria.

### Typical Efficacies of the Drugs

**Figure 4:**
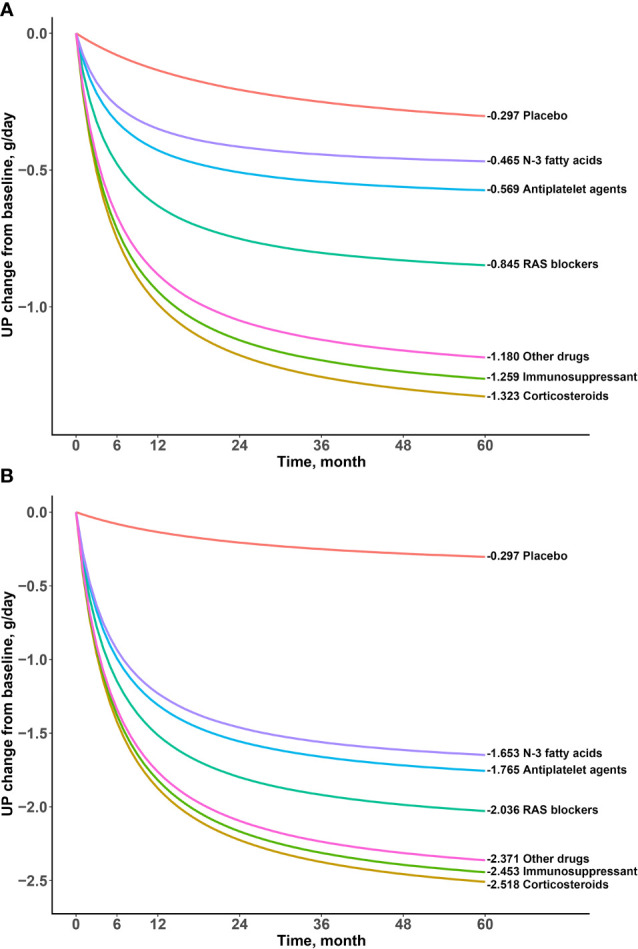
Typical time-effect curves for placebo and each drug. **(A) **Baseline urine protein was 1.80g/day **(B)** Baseline urine protein was and 3.85g/day.

**Figure 5:**
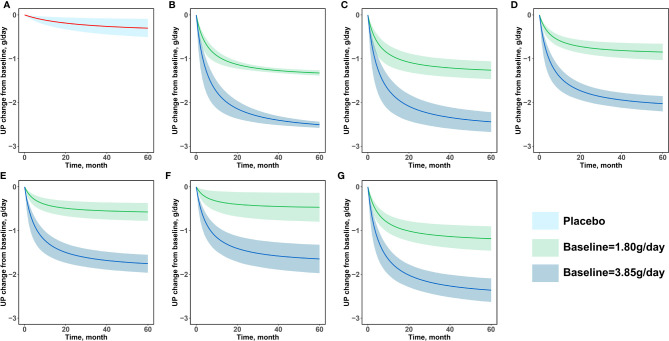
Typical values and 95% confidence intervals for placebo and individual drug effects. **(A)** Placebo **(B)** Corticosteroids **(C)** Immunosuppressants **(D)** RAS blockers **(E)** Antiplatelet agents **(F)** N-3 fatty acids **(G)** Other drugs.

Patients with proteinuria can be divided into mild proteinuria (urinary protein excretion ≤1 g/day), moderate proteinuria (urinary protein excretion 1-3 g/day) and severe proteinuria (urinary protein excretion >3 g/day) according to the KDIGO 2021 clinical practice guideline for the management of glomerular diseases (12). In order to better reflect the clinical efficacy in patients with IgA nephropathy, we simulated the typical value and 95% CI of the drug effect on patients with mild to moderate proteinuria (median baseline of proteinuria in included studies, 1.80 g/day) and patients with severe proteinuria (median baseline of proteinuria in included studies, 3.85 g/day) at different time points based on the final model (**Table 3** and **Figures 4**, **5**).

**Table 3:** Distribution of typical values for placebo and each drug effect at baseline urine protein of 1.80g/day and 3.85g/day (median, 95%Cl).

	6 month, g/day	12 month, g/day	18 month, g/day	24 month, g/day	36 month, g/day	48 month, g/day
**Baseline = 1.80g/day**						
Placebo	-0.08 (-0.16, -0.03)	-0.14 (-0.25, -0.04)	-0.18 (-0.31, -0.06)	-0.21 (-0.36, -0.06)	-0.25 (-0.43, -0.08)	-0.28 (-0.47, -0.08)
Corticosteroids	-0.75 (-0.97, -0.62)	-0.99 (-1.14, -0.88)	-1.11 (-1.21, -1.02)	-1.18 (-1.25, -1.11)	-1.26 (-1.31, -1.20)	-1.30 (-1.36, -1.24)
Immunosuppressant	-0.72 (-1.01, -0.53)	-0.94 (-1.20, -0.74)	-1.06 (-1.29, -0.85)	-1.12 (-1.34, -0.92)	-1.20 (-1.40, -0.99)	-1.24 (-1.44, -1.03)
RAS blockers	-0.48 (-0.62, -0.37)	-0.63 (-0.77, -0.50)	-0.71 (-0.85, -0.56)	-0.75 (-0.90, -0.59)	-0.80 (-0.97, -0.63)	-0.83 (-1.01, -0.65)
Antiplatelet agents	-0.32 (-0.48, -0.21)	-0.43 (-0.59, -0.28)	-0.48 (-0.65, -0.31)	-0.51 (-0.69, -0.33)	-0.54 (-0.74, -0.35)	-0.56 (-0.76, -0.36)
N-3 fatty acids	-0.27 (-0.46, -0.08)	-0.35 (-0.59, -0.10)	-0.39 (-0.66, -0.12)	-0.42 (-0.70, -0.12)	-0.44 (-0.75, -0.13)	-0.46 (-0.78, -0.14)
Other drugs	-0.67 (-0.89, -0.51)	-0.88 (-1.10, -0.68)	-0.99 (-1.21, -0.77)	-1.05 (-1.28, -0.81)	-1.12 (-1.37, -0.86)	-1.16 (-1.43, -0.89)
**Baseline = 3.85g/day**						
Placebo	-0.08 (-0.16, -0.03)	-0.14 (-0.25, -0.04)	-0.18 (-0.31, -0.06)	-0.21 (-0.36, -0.06)	-0.25 (-0.43, -0.08)	-0.28 (-0.47, -0.08)
Corticosteroids	-1.42 (-1.89, -1.15)	-1.87 (-2.21, -1.64)	-2.09 (-2.34, -1.90)	-2.23 (-2.42, -2.07)	-2.37 (-2.50, -2.27)	-2.46 (-2.55, -2.38)
Immunosuppressant	-1.38 (-1.91, -1.07)	-1.82 (-2.24, -1.52)	-2.04 (-2.39, -1.76)	-2.17 (-2.47, -1.90)	-2.31 (-2.58, -2.07)	-2.39 (-2.64, -2.17)
RAS blockers	-1.15 (-1.51, -0.93)	-1.51 (-1.79, -1.31)	-1.69 (-1.91, -1.51)	-1.80 (-2.00, -1.63)	-1.92 (-2.10, -1.75)	-1.99 (-2.16, -1.82)
Antiplatelet agents	-0.99 (-1.34, -0.78)	-1.31 (-1.59, -1.10)	-1.46 (-1.71, -1.26)	-1.56 (-1.78, -1.36)	-1.66 (-1.87, -1.46)	-1.72 (-1.93, -1.52)
N-3 fatty acids	-0.93 (-1.27, -0.70)	-1.23 (-1.54, -0.97)	-1.38 (-1.68, -1.10)	-1.46 (-1.77, -1.17)	-1.56 (-1.87, -1.25)	-1.61 (-1.93, -1.30)
Other drugs	-1.34 (-1.75, -1.08)	-1.76 (-2.09, -1.51)	-1.97 (-2.25, -1.73)	-2.10 (-2.36, -1.85)	-2.24 (-2.49, -1.98)	-2.31 (-2.57, -2.05)

The results showed that the onset time of all drugs was the same at 5.59 months, indicating a duration of 22.36 months to reach the efficacy plateau (80% of the maximum efficacy). After treatment for 6 months, 1 year, 2 years, 3 years, and 4 years, the drug efficacy reached 52%, 68%, 81%, 87%, and 90% of the maximum efficacy, respectively. In addition, we found that a higher daily urinary protein excretion at baseline was associated with better drug efficacy. For example, the efficacy of the drugs at 2 years at a baseline of 3.85 g/day was approximately 2–3 times higher than that at baseline of 1.80g/day. After deducting the effect of the baseline, the order of drug efficacy was as follows: corticosteroids > immunosuppressants > other drugs > RAS blockers > antiplatelet agents > N-3 fatty acids. When the baseline was 1.80 g/day, taking 2 years as an example, the efficacy of corticosteroids was -1.18(95% CI: -1.25, -1.11) g/day, which was significantly better than those of N-3 fatty acids (-0.42 [95% CI: -0.70, -0.12] g/day) and antiplatelet agents (-0.51 [95% CI: -0.69, -0.33] g/day).

## Discussion

At present, there are a wide variety of clinical treatment drugs for IgA nephropathy, and it is particularly important to comprehensively evaluate the difference in efficacy between various drugs for rational use in clinical treatment (23). In this study, the change in daily urinary protein excretion from baseline was selected as the efficacy indicator, and the time–effect relationship of 6 classes of drugs with different pharmacological mechanisms was quantitatively evaluated using the MBMA method. In this study, we found that there was an obvious time–effect relationship in the drug efficacy for IgA nephropathy. The shortest treatment duration included in this study was 1 month, and the longest was 48 months. The results showed that the drug efficacy reached 15% of its maximum efficacy at 1 month, while 90% of its maximum efficacy was reached at 48 months. The heterogeneity in treatment duration should be considered when estimating sample size or comparing efficacy in different studies.

This study investigated the influence of the daily urinary protein excretion at baseline, patient age, and male proportion on efficacy. The results showed that proteinuria at baseline had a significant impact on E_max_ of the drug arm, which was manifested by an increase of 0.63 g/day in E_max_ of the drug arm for every 1g/day increase in the proteinuria baseline value. Therefore, when comparing the efficacy of different drugs, it is necessary to correct for the heterogeneity in proteinuria at baseline between studies; otherwise, bias will be introduced. It should also be noted that although the drug efficacy increased when the urinary protein at baseline was high, the increase in efficacy was always less than that of the baseline; thus, urinary protein in patients with severe proteinuria was still higher than that in patients with mild to moderate proteinuria after drug treatment. For example, when proteinuria at baseline was 3.85 g/day and 1.80 g/day, 2 years after corticosteroid use, the patients’ proteinuria were approximately 1.62 g/day and 0.62 g/day, respectively, with the former still more severe than the latter.

The model established in this study was used for the quantitative analysis of the time–effect relationship of drugs and influence of proteinuria at baseline, such that the heterogeneity in the treatment course and proteinuria at baseline could be corrected for. The results showed that the drugs for the treatment of IgA nephropathy may be divided into three categories according to their efficacy: corticosteroids, immunosuppressants and other drugs had the best efficacy; RAS blockers had a moderate efficacy; and antiplatelet agents and N-3 fatty acids had the worst efficacy. For example, at 24 months, when the proteinuria at baseline was adjusted to 1.80 g/day, the most effective corticosteroids reduced urinary protein excretion by 1.18 g/day, while the least effective N-3 fatty acids reduced urinary protein excretion by 0.42 g/day, with the former being approximately 2.8 times more than the latter. When the proteinuria at baseline was adjusted to 3.85 g/day, corticosteroids and N-3 fatty acids reduced urinary protein excretion by 2.23 g/day and 1.46 g/day, respectively, with the former being approximately 1.5 times more than the latter. The results showed that the absolute difference was nearly constant as proteinuria at baseline increased, although the relative ratio of efficacy between drugs decreased gradually. For example, the difference in efficacy between corticosteroids and N-3 fatty acids remained at 0.76 g/day at 24 months, regardless of proteinuria at baseline. *In vivo*, corticosteroids bind to glucocorticoid receptors in cells, enhance kinase activity, release proteins involved in cell signaling cascades, and inhibit the occurrence of abnormal immunity (24). Despite the good efficacy of corticosteroids, the main adverse effects of corticosteroids are aggravated infection, gastric ulcers, osteoporosis, metabolic disorders, and mental disorders (25). Therefore, it is necessary to evaluate the benefit–risk ratio based on clinical practice. In addition, although the efficacy of N-3 fatty acids was found to be lower in this study, it was superior to that of the placebo, especially when the proteinuria at the baseline was higher. N-3 fatty acids act on the renal inflammatory pathway to affect the level of glomerular proteinuria and regulate cholesterol and triglycerides to delay renal toxicity associated with dyslipidemia (26). Considering that N-3 fatty acids are essential fatty acids for the human body, long-term use of N-3 fatty acids is safer than other drugs; thus, it also has a certain value for treating IgA nephropathy. This study found that the efficacy of other drugs was also remarkable. The treatment regimens of other drugs included amlodipine alone, tacrolimus alone, probucol + valsartan, mizoribine + losartan, telmisartan + clopidogrel + leflunomide, prednisolone + azathioprine + heparin + warfarin + dipyridamole, etc. Unfortunately, due to the small number of these trials, this study did not distinguish the efficacy of various regimens.

IgA nephropathy is a chronic progressive disease with a long clinical treatment course (usually 2 years or more) and self-worsening; therefore, subjects in the placebo group generally received basic treatment in clinical trials. Basic treatment aims to maintain disease progression, with lowering blood pressure as the main method. Due to the wide variety of basic treatments reported in the literature, the effects of different basic treatments on the placebo effect were not investigated in this study. This study showed that IgA nephropathy was less effective in the placebo arm, with a decrease of only 0.21 g/day in daily urinary protein excretion compared with baseline at 24 months, suggesting that the improvement in basic treatments for IgA nephropathy was small and likely only a maintenance effect. Therefore, even though there may be heterogeneity in the placebo effect between different basic treatments, the impact of heterogeneity on efficacy is low because of the small placebo effect.

The limitations of this study were as follows. The “other drugs” included 13 various treatment regimens that do not fall into the other classes. We could not analyze these treatment regimens separately because of the small number of these trials (only 1 trial arm per treatment regimens). Since most of the included literature does not distinguish between specific types of IgA nephropathy, the model established in this study may not be applicable to patients with variant forms of IgA nephropathy. The disease progression and influencing factors must be further studied and explored for variant forms of IgA nephropathy, such as minimal change disease, acute kidney injury, and rapidly progressive glomerulonephritis (12). In addition, the literature included in this study only included articles published in English, which may have resulted in publication bias.

This study was the first to comprehensively compare the efficacy of 6 classes of drugs with different pharmacological mechanisms for treating IgA nephropathy. The results showed that corticosteroids, immunosuppressants and other drugs have the best efficacy; RAS blockers have moderate efficacy; and antiplatelet agents and N-3 fatty acids have the worst efficacy. The onset times of the drugs are the same, showing an obvious time–effect relationship. In addition, this study found that proteinuria at baseline is an important factor of drug efficacy; therefore, the treatment duration and proteinuria at baseline should be corrected when comparing different studies. The results of this study provide an important reference for rational drug use for the clinical treatment of IgA nephropathy and provide a reliable reference for the optimization of clinical guidelines for IgA nephropathy.

## Data Availability Statement

The original contributions presented in the study are included in the article/**Supplementary Material**. Further inquiries can be directed to the corresponding authors.

## Author Contributions

JY: Data curation-Lead, Formal analysis-Lead, Investigation-Lead, Resources-Lead, Writing-original draft-Lead. JL, HZ, and ZS: Data curation-Supporting. HL Conceptualization-Supporting, Funding acquisition-Equal, Methodology-Supporting, Writing-review & editing-Supporting. LL Conceptualization-Lead, Funding acquisition-Lead, Methodology-Lead, Writing-review & editing-Equal. QZ Conceptualization-Lead, Formal analysis-Supporting, Funding acquisition-Supporting, Investigation-Equal, Methodology-Lead, Project administration-Equal, Software-Equal, Supervision-Equal, Validation-Equal, Visualization-Equal, Writing-original draft-Supporting, Writing-review and editing-Lead. All authors contributed to the article and approved the submitted version.

## Funding

This work was received financial support from the National Natural Science Funds (82174229) and the project of Shanghai S&T Innovation Plan (17401970900).

## Conflict of Interest

The authors declare that the research was conducted in the absence of any commercial or financial relationships that could be construed as a potential conflict of interest.

## Publisher’s Note

All claims expressed in this article are solely those of the authors and do not necessarily represent those of their affiliated organizations, or those of the publisher, the editors and the reviewers. Any product that may be evaluated in this article, or claim that may be made by its manufacturer, is not guaranteed or endorsed by the publisher.

## Abbreviations

IgA, Immunoglobulin A; RAS, renin–angiotensin system; KDIGO, Kidney Disease Improving Global Outcomes; JSN, Japanese Society of Nephrology; MBMA, Model-based meta-analysis; OBS, observed values; PRED, population predicted values; IPRED, individual predicted values; CWRES, conditional weighted residual errors.
