# Supplementary material for: Quantitative Comparison of the Clinical Efficacy of 6 Classes Drugs for IgA Nephropathy: A Model-Based Meta-Analysis of Drugs for Clinical Treatments
Source: Front Immunol. 2022 Mar 28;13:825677. doi: 10.3389/fimmu.2022.825677 (PMC9000973; doi:10.3389/fimmu.2022.825677)
Supplement: Supplementary file 2 [file Table_1.doc]

**SUPPLEMENTARY MATERIALS**

**Table S1** Search strategy

*PubMed:*

| No. | Query | Results | Dates |
| --- | --- | --- | --- |
| #1 | ( "Berger's disease" OR "immunoglobulin A nephropathy" OR "IgA nephropathy" OR "IgAN" OR "IgA nephritis" OR "IgA glomerulonephritis" ) AND ( "Captopril" OR "Ramipril" OR "Enalapril" OR "Trandolapril" OR "Benazepril" OR "Temocapril" OR "Losartan" OR "Valsartan" OR "Candesartan" OR "Olmesartan" OR "Verapamil" OR "Prednisone" OR "Prednisolone" OR "Methylprednisolone" OR "Cyclophosphamide" OR "Azathioprine" OR "Cyclosporine" OR "Mycophenolate mofetil" OR "Mizoribine" OR "Dipyridamole" OR "Warfarin" OR "Aspirin" OR "Dilazep" OR "Ticlopidine" OR "Tacrolimus" OR "RAS blockers" OR "Corticosteroids" OR "Nonsteroidal immunosuppressive agents" OR "Antiplatelet agents" OR "fish oil" ) | 173 | 18-Nov-19 |

*EMBASE:*

| No. | Query | Results | Dates |
| --- | --- | --- | --- |
| #1 | ('berger disease'/exp OR 'berger disease' OR 'immunoglobulin a nephropathy' OR 'iga nephropathy' OR 'igan' OR 'iga nephritis' OR 'iga glomerulonephritis') AND ('captopril' OR 'ramipril' OR 'enalapril' OR 'trandolapril' OR 'benazepril' OR 'temocapril' OR 'losartan' OR 'valsartan' OR 'candesartan' OR 'olmesartan' OR 'verapamil' OR 'prednisone' OR 'prednisolone' OR 'methylprednisolone' OR 'cyclophosphamide' OR 'azathioprine' OR 'cyclosporine' OR 'mycophenolate mofetil' OR 'mizoribine' OR 'dipyridamole' OR 'warfarin' OR 'aspirin' OR 'dilazep' OR 'ticlopidine' OR 'tacrolimus' OR 'fish oil') AND ([controlled clinical trial]/lim OR [randomized controlled trial]/lim) | 213 | 18-Nov-19 |

**Table S2 Summary of included studies**

| **ARMID** | **Author** | **Year** | **Country** | **Treatment** | **Class** | **Sample size** | **Mean age,**  **(year)** | **Male**  **(%)** | **Urinary protein**  **baseline**  **(g/day)** |
| --- | --- | --- | --- | --- | --- | --- | --- | --- | --- |
| 1 | Ballardie F W | 2002 | UK | Azathioprine | Immunosuppressant | 19 | NA | NA | 3.9 |
| 2 | Ballardie F W | 2002 | UK | Placebo | Placebo | 19 | NA | NA | 4.57 |
| 3 | Hollenberg N K | 2002 | Germany | Candesartan | RAS blockers | 6 | 52 | 50 | 2 |
| 4 | Hollenberg N K | 2002 | Germany | Placebo | Placebo | 6 | 52 | 50 | 1.8 |
| 5 | Chan M K | 1987 | Hong Kong | Placebo | Placebo | 19 | 27.5 | 73.7 | 1.72 |
| 6 | Chen X | 2004 | China | Urokinase+Benazepril | RAS blockers | 35 | 30.3 | 71 | 1.82 |
| 7 | Chen X | 2004 | China | Benazepril | RAS blockers | 36 | 31.3 | 64 | 1.79 |
| 8 | Cheng G | 2015 | China | Valsartan | RAS blockers | 42 | 33.9 | 63.7 | 2.4 |
| 9 | Cheng G | 2015 | China | Valsartan+Clopidogrel | Other drugs | 42 | 34.1 | 63.7 | 2.31 |
| 10 | Cheng G | 2015 | China | Valsartan+Leflunomide | Other drugs | 42 | 33.7 | 63.7 | 2.52 |
| 11 | Cheng G | 2015 | China | Valsartan+Clopidogrel+Leflunomide | Other drugs | 42 | 32.9 | 63.7 | 2.48 |
| 12 | Josep M. Cruzado | 2011 | Spain | Sirolimus+Enalapril+Atorvastatin | Other drugs | 14 | 42 | 85.7 | 2.9 |
| 13 | Josep M. Cruzado | 2011 | Spain | Enalapril+Atorvastatin | Other drugs | 9 | 50 | 77.8 | 3.7 |
| 14 | Manuel F P | 2009 | Italy | Polyunsaturated fatty acid | N-3 fatty acids | 15 | 39.5 | 46.6 | 1.31 |
| 15 | Manuel F P | 2009 | Italy | Ramipril+Irbesartan | RAS blockers | 15 | 41.5 | 73.7 | 1.45 |
| 16 | Kirmizis D | 2004 | Greece | Eicosapentaenoic acid+Docosahexaenoic acid | N-3 fatty acids | 14 | 41 | 71 | 2 |
| 17 | Kirmizis D | 2004 | Greece | Placebo | Placebo | 14 | 39 | 86 | 1.6 |
| 18 | Donadio J V | 2001 | USA | Low-dose Eicosapentaenoic acid+Docosahexaenoic acid | N-3 fatty acids | 37 | 46 | 84 | 1.79 |
| 19 | Donadio J V | 2001 | USA | High-dose Eicosapentaenoic acid+Docosahexaenoic acid | N-3 fatty acids | 36 | 45 | 81 | 1.53 |
| 20 | Gershon F | 2005 | USA | Mycophenolate | Immunosuppressant | 17 | 39 | 94.1 | 2.7 |
| 21 | Gershon F | 2005 | USA | Placebo | Placebo | 15 | 37 | 73.3 | 2.7 |
| 22 | Horita Yoshio | 2006 | Japan | Temopril | RAS blockers | 14 | 43 | 57.1 | 0.6 |
| 23 | Horita Yoshio | 2006 | Japan | Losartan | RAS blockers | 16 | 42 | 56.3 | 0.83 |
| 24 | Horita Yoshio | 2006 | Japan | Temopril+Losartan | RAS blockers | 13 | 38 | 53.8 | 0.8 |
| 25 | Horita Yoshio | 2004 | Japan | Temopril | RAS blockers | 10 | 39.6 | 40 | 0.73 |
| 26 | Horita Yoshio | 2004 | Japan | Losartan | RAS blockers | 10 | 42.7 | 50 | 0.81 |
| 27 | Horita Yoshio | 2004 | Japan | Temopril+Losartan | RAS blockers | 11 | 39.6 | 45.5 | 0.75 |
| 28 | Horita Yoshio | 2007 | Japan | Prednisolone+Losartan | Other drugs | 20 | 34 | 60 | 1.6 |
| 29 | Horita Yoshio | 2007 | Japan | Prednisolone | Corticosteroids | 18 | 32 | 44.4 | 1.6 |
| 30 | Lai K N | 1991 | Hong Kong | Cyclosporine A | Immunosuppressant | 11 | 33.3 | 36.4 | 3.8 |
| 31 | Lai K N | 1991 | Hong Kong | Placebo | Placebo | 11 | 37.7 | 54.5 | 2.6 |
| 32 | Liu Xiaowei | 2014 | China | Mycophenolate | Immunosuppressant | 42 | 39.8 | 57.1 | 2.83 |
| 33 | Liu Xiaowei | 2014 | China | Cyclophosphamide | Immunosuppressant | 42 | 37.4 | 64.3 | 2.77 |
| 34 | Nakamura Tsukasa | 2007 | Japan | Olmesartan | RAS blockers | 8 | 34 | 62.5 | 2.1 |
| 35 | Nakamura Tsukasa | 2007 | Japan | Temopril | RAS blockers | 8 | 31 | 50 | 2 |
| 36 | Nakamura Tsukasa | 2007 | Japan | Olmesartan+Temopril | RAS blockers | 8 | 31 | 50 | 1.9 |
| 37 | Cheon P H | 2003 | Korea | Losartan | RAS blockers | 20 | 39.3 | 45 | 2.3 |
| 38 | Cheon P H | 2003 | Korea | Amlodipine | Other drugs | 16 | 44.3 | 56.3 | 2.1 |
| 39 | Perico N | 1998 | Italy | Enalapril | RAS blockers | 11 | 31 | 81.8 | 1.51 |
| 40 | Perico N | 1998 | Italy | Irbesartan | RAS blockers | 9 | 46 | 77.8 | 2.38 |
| 41 | Remuzzi A | 1999 | Italy | Enalapril | RAS blockers | 11 | NA | NA | 1.44 |
| 42 | Remuzzi A | 1999 | Italy | Irbesartan | RAS blockers | 9 | NA | NA | 2.48 |
| 43 | Segarra Alfons | 2007 | Spain | Mycophenolate | Immunosuppressant | 25 | 37 | 76 | 2.74 |
| 44 | Shen Pei-Cheng | 2012 | China | Losartan | RAS blockers | 112 | 50.2 | 51.8 | 1.72 |
| 45 | Shen Pei-Cheng | 2012 | China | Placebo | Placebo | 114 | 49.1 | 49.1 | 1.73 |
| 46 | Shimizu Ari | 2008 | Japan | Losartan | RAS blockers | 18 | 36 | 61.1 | 0.81 |
| 47 | Shimizu Ari | 2008 | Japan | Placebo | Placebo | 18 | 35.7 | 33.3 | 0.73 |
| 48 | Shoji T | 2000 | Japan | Prednisolone | Corticosteroids | 11 | 28.7 | 45.5 | 0.75 |
| 49 | Shoji T | 2000 | Japan | Dipyridamole | Antiplatelet agents | 8 | 33.3 | 12.5 | 0.73 |
| 50 | Suzuki Takayuki | 2004 | Japan | Prednisolone | Corticosteroids | 6 | 33.8 | 66.6 | 1.2 |
| 51 | Suzuki Takayuki | 2004 | Japan | Prednisolone | Corticosteroids | 21 | 24.7 | 61.9 | 1.09 |
| 52 | Tomino Y | 1987 | Japan | Danazol | Other drugs | 18 | 30.7 | NA | 1.03 |
| 53 | Tomino Y | 1987 | Japan | Dipyridamole | Antiplatelet agents | 20 | NA | NA | 0.92 |
| 54 | Trimarchi Hernán | 2007 | Argentina | Methylprednisolone+Enalapril+Valsartan | Other drugs | 20 | 37.5 | 50 | 2.2 |
| 55 | Wang Zhen | 2017 | Japan | Tripterygium wilfordii | Immunosuppressant | 16 | 32.5 | 50 | 5.02 |
| 56 | Wang Zhen | 2017 | Japan | Double Tripterygium wilfordii | Immunosuppressant | 18 | 33.6 | 55.6 | 5.29 |
| 57 | Woo K T | 2000 | Singapore | Enalapril+Losartan | RAS blockers | 21 | 39 | 57.1 | 2.2 |
| 58 | Woo K T | 2000 | Singapore | Placebo | Placebo | 20 | 37 | 75 | 2.1 |
| 59 | Xie Yuansheng | 2011 | China | Losartan | RAS blockers | 30 | 33.7 | 46.7 | 1.12 |
| 60 | Xie Yuansheng | 2011 | China | Mizoribine | Immunosuppressant | 35 | 33.6 | 40 | 1.35 |
| 61 | Xie Yuansheng | 2011 | China | Losartan+Mizoribine | Other drugs | 34 | 33.7 | 41.2 | 1.21 |
| 62 | Xu Lin | 2013 | China | Prednisone | Corticosteroids | 48 | 34.5 | 58.3 | 2.01 |
| 63 | Xu Lin | 2013 | China | Cyclosporine A | Immunosuppressant | 48 | 35.4 | 50 | 2.07 |
| 64 | Ye Zhiming | 2014 | China | Probucol+Valsartan | Other drugs | 33 | 34 | 36.4 | 1.39 |
| 65 | Ye Zhiming | 2014 | China | Valsartan | RAS blockers | 35 | 34 | 42.9 | 1.47 |
| 66 | Konishi Yoshio | 2008 | Japan | Prednisolone+Valsartan | Other drugs | 9 | 25 | 33.3 | 0.94 |
| 67 | Konishi Yoshio | 2008 | Japan | Prednisolone | Corticosteroids | 9 | 27 | 11.1 | 0.57 |
| 68 | Li Philip Kam-Tao | 2006 | Hong Kong | Valsartan | RAS blockers | 54 | 40 | 24.1 | 1.8 |
| 69 | Li Philip Kam-Tao | 2006 | Hong Kong | Placebo | Placebo | 55 | 41 | 30.9 | 2.35 |
| 70 | Maes Bart D | 2004 | Belgium | Mycophenolate | Immunosuppressant | 21 | NA | NA | 1.9 |
| 71 | Maes Bart D | 2004 | Belgium | Placebo | Placebo | 13 | NA | NA | 1.3 |
| 72 | Manno Carlo | 2009 | Italy | Ramipril | RAS blockers | 49 | 34.9 | 71.4 | 1.5 |
| 73 | Manno Carlo | 2009 | Italy | Ramipril+Prednisone | Other drugs | 48 | 31.8 | 68.8 | 1.7 |
| 74 | Praga M | 2003 | Spain | Enalapril | RAS blockers | 23 | 27.8 | 65 | 2 |
| 75 | Praga M | 2003 | Spain | Placebo | Placebo | 21 | 29.9 | 57 | 1.7 |
| 76 | Pozzi Claudio | 2013 | Italy | Azathioprine | Immunosuppressant | 20 | 43 | 85 | 3.21 |
| 77 | Pozzi Claudio | 2013 | Italy | Prednisone | Corticosteroids | 26 | 37.7 | 77 | 1.99 |
| 78 | Pozzi Claudio | 2010 | Italy | Azathioprine | Immunosuppressant | 101 | 34.8 | 75 | 2.09 |
| 79 | Pozzi Claudio | 2010 | Italy | Prednisone | Corticosteroids | 106 | 40.5 | 71 | 1.96 |
| 80 | Pozzi Claudio | 2004 | Italy | Placebo | Placebo | 43 | 40 | 72.1 | 1.92 |
| 81 | Pozzi Claudio | 2004 | Italy | Prednisone | Corticosteroids | 43 | 38 | 69.8 | 2.14 |
| 82 | Donadio J V | 1999 | USA | Eicosapentaenoic acid+Docosahexaenoic acid | N-3 fatty acids | 55 | 37 | 74 | 2.55 |
| 83 | Donadio J V | 1999 | USA | Placebo | Placebo | 51 | 37 | 73 | 3.22 |

**Reference S1** List of included studies

1. Ballardie F W, Roberts I. Controlled prospective trial of prednisolone and cytotoxics in progressive IgA nephropathy. Journal of the American Society of Nephrology, 2002, 13(1):142-148.
2. Hollenberg N K. Reduction of proteinuria; combined effects of receptor blockade and low dose angiotensin-converting enzyme inhibition. Journal of Hypertension, 2002, 20(4):739-743.
3. Chan M K, Kwan S, Chan K W, et al. Controlled trial of antiplatelet agents in mesangial IgA glomerulonephritis. American Journal of Kidney Diseases, 1987, 9(5):417-421.
4. Chen X, Qiu Q, Tang L, et al. Effects of co-administration of urokinase and benazepril on severe IgA nephropathy. Nephrology, dialysis, transplantation: official publication of the European Dialysis and Transplant Association - European Renal Association, 2004(4):852.
5. Cheng G, Liu D, Margetts P, et al. Valsartan combined with clopidogrel and/or leflunomide for the treatment of progressive immunoglobulin A nephropathy. Nephrology, 2015, 20(2):77-84.
6. J, M, Cruzado, et al. Low-dose sirolimus combined with angiotensin-converting enzyme inhibitor and statin stabilizes renal function and reduces glomerular proliferation in poor prognosis IgA nephropathy[J]. Nephrology Dialysis Transplantation, 2011.
7. Manuel F P, Franco F G, Giovanni G, et al. Combined treatment with renin-angiotensin system blockers and polyunsaturated fatty acids in proteinuric IgA nephropathy: a randomized controlled trial. Nephrology, dialysis, transplantation: official publication of the European Dialysis and Transplant Association-European Renal Association, 2009(1):156-60.
8. Kirmizis D. Treatment of severe IgA nephropathy with omega-3 fatty acids: the effect of a "very low dose" regimen. ren fail, 2004, 26(4):453-459.
9. Donadio J V, Larson T S, Bergstralh E J. A Randomized Trial of High-Dose Compared with Low-Dose Omega-3 Fatty Acids in Severe IgA Nephropathy. Journal of the American Society of Nephrology, 2001, 12(4):791-799.
10. Gershon F, Lin J, Jordan R, et al. Mycophenolate mofetil (MMF) vs placebo in patients with moderately advanced IgA nephropathy: a double-blind randomized controlled trial. Nephrology Dialysis Transplantation, 2005(10):2139-2145.
11. Horita Yoshio, Taura Kouichi, Taguchi Takashi et al. Aldosterone breakthrough during therapy with angiotensin-converting enzyme inhibitors and angiotensin II receptor blockers in proteinuric patients with immunoglobulin A nephropathy. Nephrology (Carlton), 2006, 11: 462-6.
12. Horita Yoshio, Tadokoro Masato, Taura Koichi et al. Low-dose combination therapy with temocapril and losartan reduces proteinuria in normotensive patients with immunoglobulin a nephropathy. Hypertens Res, 2004, 27: 963-70.
13. Horita Yoshio, Tadokoro Masato, Taura Kouichi et al. Prednisolone co-administered with losartan confers renoprotection in patients with IgA nephropathy. Ren Fail, 2007, 29: 441-6.
14. Lai K N, Lam C W, Cheng I K et al. Effect of cyclosporine A on circulating immune complexes in IgA nephropathy. Int Urol Nephrol, 1991, 23: 265-74.
15. Liu Xiaowei, Dewei Du, Sun Shiren et al. Treatment of severe IgA nephropathy: mycophenolate mofetil/prednisone compared to cyclophosphamide/prednisone. Int J Clin Pharmacol Ther, 2014, 52: 95-102.
16. Nakamura Tsukasa, Inoue Teruo, Sugaya Takeshi et al. Beneficial effects of olmesartan and temocapril on urinary liver-type fatty acid-binding protein levels in normotensive patients with immunoglobin A nephropathy. Am J Hypertens, 2007, 20: 1195-201.
17. Cheon P H, Xu Z G, Sorae C, et al. Effect of losartan and amlodipine on proteinuria and transforming growth factor-beta1 in patients with IgA nephropathy. Nephrol Dial Transplant, 2003(6):1115-1121.
18. Perico N, Remuzzi A, Sangalli F et al. The antiproteinuric effect of angiotensin antagonism in human IgA nephropathy is potentiated by indomethacin. J Am Soc Nephrol, 1998, 9: 2308-17.
19. Remuzzi A, Perico N, Sangalli F et al. ACE inhibition and ANG II receptor blockade improve glomerular size-selectivity in IgA nephropathy. Am J Physiol, 1999, 276: F457-66.
20. Segarra Alfons, Amoedo Ma Luisa, Martinez Garcia Jose Ma et al. Efficacy and safety of 'rescue therapy' with mycophenolate mofetil in resistant primary glomerulonephritis--a multicenter study. Nephrol Dial Transplant, 2007, 22: 1351-60.
21. Shen Pei-Cheng, He Li-Qun, Yang Xue-Jun et al. Renal protection of losartan 50 mg in normotensive Chinese patients with nondiabetic chronic kidney disease. J Investig Med, 2012, 60: 1041-7.
22. Shimizu Ari, Takei Takashi, Uchida Keiko et al. Low-dose losartan therapy reduces proteinuria in normotensive patients with immunoglobulin A nephropathy. Hypertens Res, 2008, 31: 1711-7.
23. Shoji T, Nakanishi I, Suzuki A et al. Early treatment with corticosteroids ameliorates proteinuria, proliferative lesions, and mesangial phenotypic modulation in adult diffuse proliferative IgA nephropathy. Am J Kidney Dis, 2000, 35: 194-201.
24. Suzuki Takayuki, Yamamoto Tatsuo, Ohura Masaharu et al. Clinicopathologic findings relevant to disappearance or relapse of proteinuria following corticosteroid treatment in IgA nephropathy patients with proteinuria of 0.5 to 2.0 g/day. Clin Exp Nephrol, 2004, 8: 243-9.
25. Tomino Y, Sakai H, Hanzawa S et al. Clinical effect of danazol in patients with IgA nephropathy. Jpn J Med, 1987, 26: 162-6.
26. Trimarchi Hernán, Muryan Alexis, Young Pablo et al. Dual renin-angiotensin system blockade plus oral methylprednisone for the treatment of proteinuria in IgA nephropathy. Medicina (B Aires), 2007, 67: 445-50.
27. Wang Zhen, Yu Chao, Zhou Li-Na et al. Effects of Tripterygium wilfordii Induction Therapy to IgA Nephropathy Patients with Heavy Proteinuria. Biol Pharm Bull, 2017, 40: 1833-1838.
28. Woo K T, Lau Y K, Wong K S et al. ACEI/ATRA therapy decreases proteinuria by improving glomerular permselectivity in IgA nephritis. Kidney Int, 2000, 58: 2485-91.
29. Xie Yuansheng, Huang Songmin, Wang Li et al. Efficacy and safety of mizoribine combined with losartan in the treatment of IgA nephropathy: a multicenter, randomized, controlled study. Am J Med Sci, 2011, 341: 367-72.
30. Xu Lin, Liu Zhong-Cheng, Guan Guang-Ju et al. Cyclosporine A combined with medium/low dose prednisone in progressive IgA nephropathy. Kaohsiung J Med Sci, 2014, 30: 390-5.
31. Ye Zhiming, Zhang Li, Xu Lixia et al. Probucol combined with valsartan in immunoglobulin A nephropathy: a multi-centre, open labelled, randomized controlled study. Nephrology (Carlton), 2014, 19: 40-6.
32. Konishi Yoshio, Morikawa Takashi, Okada Noriyuki et al. Evidence for abundant presence of chymase-positive mast cells in the kidneys of patients with immunoglobulin A nephropathy: effect of combination therapy with prednisolone and angiotensin II receptor blocker valsartan. Hypertens Res, 2008, 31: 1517-24.
33. Li Philip Kam-Tao, Leung Chi Bon, Chow Kai Ming et al. Hong Kong study using valsartan in IgA nephropathy (HKVIN): a double-blind, randomized, placebo-controlled study. Am J Kidney Dis, 2006, 47: 751-60.
34. Maes Bart D, Oyen Raymond, Claes Kathleen et al. Mycophenolate mofetil in IgA nephropathy: results of a 3-year prospective placebo-controlled randomized study. Kidney Int, 2004, 65: 1842-9.
35. Manno Carlo, Torres Diletta Domenica, Rossini Michele et al. Randomized controlled clinical trial of corticosteroids plus ACE-inhibitors with long-term follow-up in proteinuric IgA nephropathy. Nephrol Dial Transplant, 2009, 24(12): 3694-701.
36. Praga M, Hernandez E, Gonzalez E, et al. Treatment of IgA nephropathy with ACE inhibitors: a randomized and controlled trial. Journal of the American Society of Nephrology, 2003, 14(6):1578-1583.
37. Pozzi Claudio, Andrulli Simeone, Pani Antonello et al. IgA nephropathy with severe chronic renal failure: a randomized controlled trial of corticosteroids and azathioprine. J Nephrol, 2013, 26(1): 86-93.
38. Pozzi C, Andrulli S, Pani A, et al. Addition of azathioprine to corticosteroids does not benefit patients with IgA nephropathy. Journal of the American Society of Nephrology, 2010, 21(10):1783-1790.
39. Pozzi C, Andrulli S, Vecchio L D, et al. Corticosteroid Effectiveness in IgA Nephropathy: Long-Term Results of a Randomized, Controlled Trial. Journal of the American Society of Nephrology, 2004, 15(1):157-163.
40. Donadio J V, Grande J P, Bergstralh E J, et al. The long-term outcome of patients with IgA nephropathy treated with fish oil in a controlled trial. Mayo Nephrology Collaborative Group. Journal of the American Society of Nephrology, 1999, 10(8):1772-1777.

**Table S3** The quality of randomized controlled trials accessed by Cochrane risk-of-bias criteria.

| **Studies** | **Random sequence generation** | **Allocation concealment** | **Blinding of participants and personnel** | **Blinding of outcomes assessment** | **Incomplete outcome data** | **Selective reporting** | **Other bias** |
| --- | --- | --- | --- | --- | --- | --- | --- |
| **Ballardie F W, 2002** | Low risk | Low risk | Unclear | High risk | Low risk | Low risk | Low risk |
| **Hollenberg N K, 2002** | Low risk | Low risk | Low risk | Low risk | Low risk | Low risk | Low risk |
| **Chan M K, 1987** | Low risk | Low risk | Low risk | Low risk | Low risk | Low risk | Low risk |
| **Chen X, 2004** | Low risk | Low risk | Unclear | High risk | Low risk | Low risk | Low risk |
| **Cheng G, 2015** | Low risk | Low risk | Low risk | Low risk | Low risk | Low risk | Low risk |
| **Josep M. Cruzado, 2011** | Low risk | Low risk | Low risk | Low risk | Low risk | Low risk | Low risk |
| **Manuel F P, 2009** | Low risk | Low risk | Low risk | Unclear | Low risk | Low risk | Low risk |
| **Kirmizis D, 2004** | Low risk | Low risk | Unclear | Unclear | Low risk | Low risk | Low risk |
| **Donadio J V, 2001** | Low risk | Unclear | Unclear | High risk | Low risk | Low risk | Low risk |
| **Gershon F, 2005** | Low risk | Low risk | High risk | High risk | Low risk | Low risk | Low risk |
| **Horita Yoshio, 2006** | Low risk | Unclear | High risk | Unclear | Low risk | Low risk | Low risk |
| **Horita Yoshio, 2004** | Low risk | Unclear | High risk | Unclear | Low risk | Low risk | Low risk |
| **Horita Yoshio, 2007** | Low risk | Unclear | High risk | Unclear | Low risk | Low risk | Low risk |
| **Lai K N, 1991** | Low risk | Low risk | Unclear | Unclear | Low risk | Low risk | Low risk |
| **Liu Xiaowei, 2014** | Low risk | Low risk | Unclear | Unclear | Low risk | Low risk | Low risk |
| **Nakamura Tsukasa, 2007** | Low risk | Low risk | High risk | Unclear | Low risk | Low risk | Low risk |
| **Cheon P H, 2003** | Low risk | Low risk | High risk | Unclear | Low risk | Low risk | Low risk |
| **Perico N, 1998** | Low risk | Low risk | Low risk | Low risk | Low risk | Low risk | Low risk |
| **Remuzzi A, 1999** | Low risk | Low risk | Low risk | Low risk | Low risk | Low risk | Low risk |
| **Shen Pei-Cheng, 2012** | Low risk | Low risk | High risk | High risk | Low risk | Low risk | Low risk |
| **Shimizu Ari, 2008** | Low risk | Low risk | High risk | Unclear | Low risk | Low risk | Low risk |
| **Shoji T, 2000** | Low risk | Low risk | Unclear | Unclear | Low risk | Low risk | Low risk |
| **Woo K T, 2000** | Low risk | Low risk | Unclear | Unclear | Low risk | Low risk | Low risk |
| **Xie Yuansheng, 2011** | Low risk | Unclear | High risk | High risk | Low risk | Low risk | Low risk |
| **Ye Zhiming, 2014** | Low risk | Unclear | High risk | High risk | Low risk | Low risk | Low risk |
| **Li Philip Kam-Tao, 2006** | Low risk | Low risk | Low risk | Low risk | Low risk | Low risk | Low risk |
| **Maes Bart D, 2004** | Low risk | Low risk | Unclear | Unclear | Low risk | Low risk | Low risk |
| **Manno C, 2009** | Low risk | Low risk | High risk | Low risk | Low risk | Low risk | Low risk |
| **Praga M, 2003** | Low risk | Low risk | High risk | Low risk | Low risk | Low risk | Low risk |
| **Pozzi C, 2013** | Unclear | Unclear | High risk | Low risk | Low risk | Low risk | Low risk |
| **Pozzi C, 2010** | Low risk | Unclear | High risk | Low risk | Unclear | Low risk | Low risk |
| **Pozzi C, 2004** | Low risk | Unclear | High risk | Low risk | Low risk | Low risk | Low risk |
| **Donadio J V, 1999** | Unclear | Unclear | High risk | Low risk | Unclear | Low risk | Low risk |

**Table S4 The quality of the other trials accessed by NOS criteria.**

| **Studies** | **Selection** | | | | | | | | **Comparability** | **Outcome** | | | **Total score** | |
| --- | --- | --- | --- | --- | --- | --- | --- | --- | --- | --- | --- | --- | --- | --- |
|  | Representative-  ness of the exposed cohort | | Selection of the non exposed cohort | | Ascertainment of exposure | Demonstration that outcome of interest was not present at start of study | | Comparability of cohorts on the basis of the design or analysis | | Assessment of outcome | Was follow-up long enough for outcomes to occur | Adequacy of follow up of cohorts |  |  |
| **Segarra A, 2007** | ☆ | ☆ | | ☆ | | | ☆ | | ☆ | ☆ | - | ☆ | 7 | |
| **Suzuki T, 2004** | ☆ | ☆ | | ☆ | | | ☆ | | ☆ | ☆ | ☆ | ☆ | 8 | |
| **Tomino Y, 1987** | ☆ | ☆ | | ☆ | | | ☆ | | - | - | - | - | 4 | |
| **Trimarchi H, 2007** | ☆ | ☆ | | ☆ | | | ☆ | | ☆☆ | ☆ | ☆ | ☆ | 9 | |
| **Wang Z, 2017** | ☆ | ☆ | | ☆ | | | ☆ | | ☆☆ | ☆ | - | ☆ | 8 | |
| **Xu L, 2013** | ☆ | ☆ | | ☆ | | | ☆ | | ☆☆ | ☆ | ☆ | ☆ | 9 | |
| **Konishi Y, 2008** | ☆ | ☆ | | ☆ | | | ☆ | | ☆☆ | ☆ | - | - | 7 | |

☆ = one score
